# Optimal Design of Electromagnetic Absorber Based on Magnetic Field Directional Consistency

**DOI:** 10.3390/ma19030489

**Published:** 2026-01-26

**Authors:** Juan Wang, Jingjun Lou, Qingchao Yang, Ronghua Li, Maoting Tan, Ming Yang, Xu Bao

**Affiliations:** Naval University of Engineering, Wuhan 430033, China

**Keywords:** electromagnetic vibration absorber, dual-effect model, geometric optimization, magnetic field directional consistency, electromagnetic force

## Abstract

This study proposes a permanent magnet edge topology optimization method to address the low force output efficiency in Electromagnetic Vibration Absorbers caused by magnetic field distortion at the geometric discontinuity between the permanent magnet and the magnetic yoke. A theoretical model incorporating magnetic flux enhancement and improved directional consistency is established, revealing a transition in dominant mechanisms: magnetic flux enhancement dominates at small chamfer depths, while improved directional consistency becomes predominant at larger chamfer depths. Experimental validation was conducted on the optimized structure with a standard chamfer angle and 4 mm chamfer depth under operational conditions. Test results show an average force output increase of 4.6%, reaching 6.8% at 5 A. This research provides a theoretical basis and design methodology for geometric optimization of electromagnetic actuators in space-constrained environments.

## 1. Introduction

Electromagnetic Vibration Absorbers (EVAs) have become key actuators in active vibration control due to their fast response, high control precision, and robust structure [1,2,3]. In space-constrained applications such as ships, vibration control systems face dual challenges of limited installation space and increasing performance requirements. Megan F et al. [4] quantified underwater radiated noise from various merchant ships, emphasizing the importance of vibration and noise reduction. Studies on the optimization of ship cabin space [5,6] indicate that maximizing electromagnetic force output within limited installation dimensions is a critical design issue for EVAs.

Conventional optimization for EVAs primarily targets maximizing flux density. This is achieved by improving material properties [7] and refining magnetic circuit designs, with the core objective being to increase air-gap flux density and thereby enhance electromagnetic force output [8,9]. In structural innovation, SI et al. [10] analyzed static magnetic field distribution characteristics of hybrid rotor structures, while Zhao et al. [11] and Luo et al. [12] achieved decoupling of electric and magnetic loadings in transverse flux linear motors, providing new insights for thrust density improvement. To evaluate structural optimization effects, the equivalent magnetic circuit method is widely used due to its computational efficiency and parametric flexibility [13,14,15]. However, traditional equivalent magnetic circuit methods [16,17] struggle to accurately describe flux distribution characteristics in complex geometries. To address this, Huang et al. [18] and Fan et al. [19] significantly improved calculation accuracy by introducing magnetic circuit saturation correction and flux distribution coefficients. In optimization strategies, the Taguchi method [20] and response surface methodology combined with intelligent algorithms [21] have successfully achieved multi-objective optimization. Sun et al. [22] proposed a virtual winding-based analysis method and optimized drive strategy to address inductance imbalance and magnetic field distortion caused by segmented excitation in linear synchronous motors. These studies have established a comprehensive methodology for electromagnetic device performance enhancement.

However, whether using analytical methods based on equivalent magnetic circuits or numerical approaches relying on finite elements, most existing studies treat the magnetic field as a scalar quantity for “intensity” optimization. A key attribute of the magnetic field as a vector field—directional consistency—is not sufficiently addressed [23,24]. In the geometrically discontinuous regions between permanent magnets and magnetic yokes, magnetic induction lines undergo local concentration and sharp bending due to abrupt path changes, resulting in magnetic field distribution distortion [25]. He [26] revealed the effects of permanent magnet shape design and manufacturing processes on magnetic field non-uniformity from the perspectives of molecular current and demagnetizing field, while Guo et al. [27] elucidated the microscopic mechanisms of magnetic field distortion caused by stress concentration regions through multi-scale simulations. In large-scale electromagnetic devices, the impact of magnetic field distortion is more pronounced. Kang et al. [25] and Li et al. [28] found, in turbine generator studies, that the combined effects of magnetic circuit saturation and field distortion not only alter the symmetry of flux density waveforms but also affect the accuracy of inductance parameter calculations. Kim et al. [29] further pointed out that magnetic field distortion induced by current phase angle variations changes the distribution characteristics of tangential and normal magnetic forces, exacerbating electromagnetic vibration. These studies reveal that the fundamental detriment of magnetic field distortion lies in disrupting the ordered directionality of the magnetic field vectors. This disruption causes the generated electromagnetic force to deviate from the target actuation axis, which severely reduces the effective working efficiency of the force [30,31,32].

To address magnetic field distribution non-uniformity, some studies have explored geometric optimization methods. Jia et al. [33] significantly improved performance in magnetic field modulation motors by optimizing modulation unit geometric parameters, demonstrating the feasibility of geometric optimization for improving magnetic field distribution. However, existing geometric optimization methods primarily target circumferential magnetic field modulation in rotating motors, lacking systematic investigation of magnetic field distortion caused by axial geometric discontinuity in linear EVA [26,34]. According to Maxwell stress tensor theory, electromagnetic force depends not only on flux density magnitude but also on the directional distribution of the magnetic field vector. When the magnetic field direction deviates from the target actuation direction, undesired tangential force components are generated while reducing force output in the effective actuation direction.

Current research on electromagnetic force optimization using Maxwell’s stress tensor primarily employs finite element analysis to evaluate force characteristics [35] or applies tensor calculations for pole shape design to mitigate vibration [36]. Typically, however, stress tensor analysis serves only as a post-design verification tool rather than guiding the optimization process from the outset. Furthermore, existing methods lack a quantitative criterion based on magnetic field directional consistency, making it difficult to distinguish the contributions of increased field strength from improved field alignment. This issue is compounded by the scalar nature of traditional magnetic circuit analysis, which cannot describe directional properties, leaving a theoretical gap between circuit models and stress tensor methods.

This study makes three key contributions. It introduces the force directional consistency coefficient (η) as a quantitative metric, turning field direction optimization into a concrete design objective. Second, a unified model is established, combining magnetic circuit analysis with Maxwell’s stress tensor method to separately quantify improvements from flux enhancement and directional alignment. Third, using this model, the research shows how geometric parameters determine which effect dominates, providing forward design guidelines rather than mere post-evaluation. This framework offers a reusable analytical approach for optimizing electromagnetic actuators.

The paper is organized as follows: Section 2 establishes the theoretical analysis model, Section 3 conducts finite element simulation analysis, Section 4 presents experimental validation, and Section 5 summarizes the work and discusses future research directions.

## 2. Theoretical Analysis

To systematically analyze the effect of permanent magnet chamfering on EVA performance, this section establishes theoretical models from two perspectives: magnetic circuit analysis and Maxwell stress tensor analysis, and constructs a unified performance evaluation framework.

### 2.1. System Structure and Geometric Parameters

**Figure 1:**
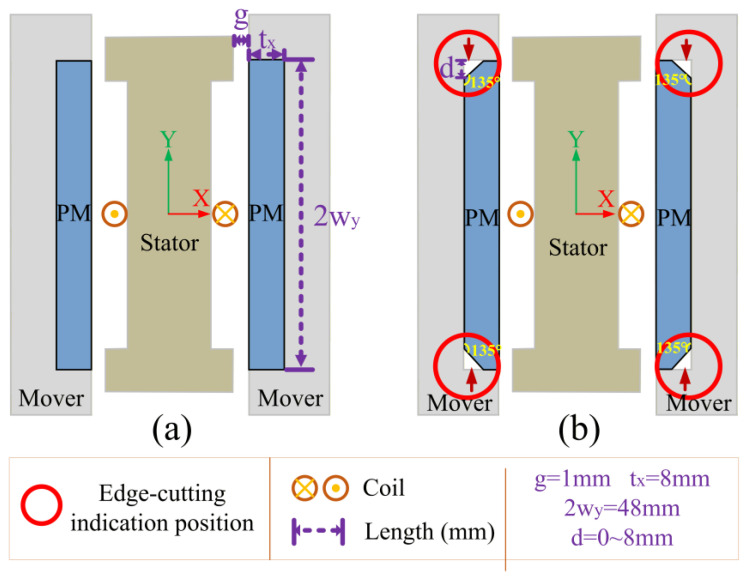
Schematic diagram of electromagnetic vibration absorber core structure (**a**) without chamfer; (**b**) with chamfer. Red circles indicate chamfer regions of interest, purple characters indicate geometric dimensions.

The core structure of the EVA under study is shown in Figure 1. The structure adopts a fully symmetric layout in all directions (up–down and left–right), where the Y-axis is defined as the vertical motion direction of the mover (the linearly oscillating component). Key geometric design parameters of the system include the following: permanent magnet thickness $t_{x}$ in the X-direction, length $w_{y}$, in the Y-direction, working air gap width g between the mover and stator, and the permanent magnet chamfer depth d, which is the focus of this study, with 0 < d < $t_{x}$. The permanent magnet chamfer location and shape are indicated in Figure 1. Unless otherwise specified, chamfering mentioned in this paper specifically refers to the geometric chamfering treatment of permanent magnets.

### 2.2. Magnetic Circuit Analysis as Follows: Flux Enhancement Quantification

**Figure 2:**
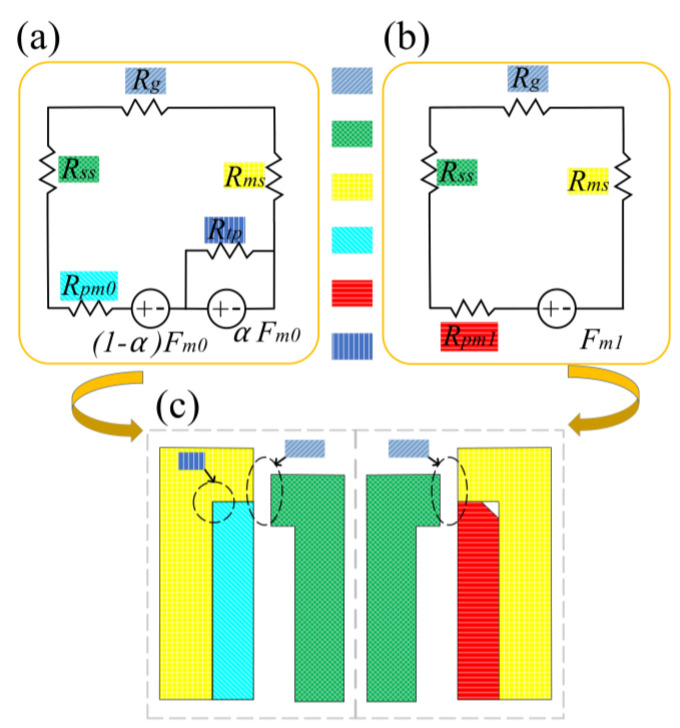
Equivalent magnetic circuit diagram (**a**) before chamfering; (**b**) after chamfering; (**c**) equivalent reluctances corresponding to quarter-structure reference, colored blocks represent corresponding reluctance regions. Blue diagonal stripes: Air gap reluctance ($R_{g}$); Green grid: Stator silicon steel reluctance ($R_{ss}$); Yellow grid: Mover silicon steel reluctance ($R_{ms}$); Cyan diagonal stripes: Permanent magnet reluctance ($R_{pm0}$); Red horizontal stripes: Permanent magnet reluctance after chamfering ($R_{pm1}$); Blue vertical stripes: Equivalent concentrated corner reluctance ($R_{tp}$).

Based on the structural symmetry, a quarter-structure equivalent magnetic circuit model is established for analysis, as shown in Figure 2.

In this equivalent magnetic circuit model, $F_{m0}$ and $F_{m1}$ represent the magnetomotive forces of the permanent magnet before and after chamfering, respectively; $R_{pm0}$ and $R_{pm1}$ represent the equivalent reluctances of the permanent magnet in the corresponding states; $R_{ms}$ represents the reluctance of the silicon steel portion in the mover; $R_{g}$ represents the reluctance of the working air gap; $R_{ss}$ represents the reluctance of the silicon steel portion in the stator.

At the geometric interface between the permanent magnet and the silicon steel mover, the magnetic field distribution becomes distorted due to abrupt structural changes, causing significant bending of magnetic induction lines. The magnetic flux path changes sharply in this region, forcing field lines to complete directional transitions within a limited space, forming localized magnetic field concentration and high-reluctance zones. Consequently, the equivalent reluctance in this corner region is significantly higher than that in straight magnetic conductive segments. For theoretical analysis, this complex distributed parameter effect is modeled as a concentrated corner reluctance $R_{tp}$ in the equivalent magnetic circuit. The equivalent reluctance quarter-structure reference is shown in Figure 2c.

According to the classical magnetomotive force balance theorem [37], under conditions where other secondary factors are neglected, the total magnetomotive force generated by the permanent magnet is distributed into two paths. One part establishes the effective working main flux, while the other part is consumed by the equivalent reluctance at the corner. In practical electromagnetic structural design, the additional magnetomotive force consumption caused by flux concentration in corner regions should be strictly controlled at a low level. Let α be the proportion of magnetomotive force consumed by corner reluctance $R_{tp}$, where 0 < α < 0.3, a range referenced from typical motor and transformer design practices [38]. The effective magnetomotive force for the corner shunt path is ${\alpha F}_{m0}$, and the effective magnetomotive force for the main magnetic circuit is $\left(1-\alpha \right)F_{m0}$.

The equivalent permanent magnet magnetomotive force $F_{m}$ is primarily determined by its residual magnetic flux density $B_{r}$ and the effective length $t_{x}$ in the magnetization direction, where $\mu_{1}$ is the relative permeability of the permanent magnet material:

(1) $$F_{m}=\frac{B_{r}t_{x}}{\mu_{1}}$$

The equivalent permanent magnet magnetomotive force $F_{m}$ is primarily determined by the material’s intrinsic characteristics and its overall length along the magnetization direction. Since chamfering modifies only the local magnetization length at the edges and has minimal influence on this overall length, $F_{m}$ before and after chamfering can be considered constant.

(2) $$F_{m0}=F_{m1}=F_{m}.$$

According to the fundamental definition of reluctance, the equivalent reluctance $R_{pm0}$ of the original permanent magnet can be expressed as follows:

(3) $$R_{pm0}=\frac{t_{x}}{\mu_{1}w_{y}L_{z}}$$

$L_{z}$ is the length of the permanent magnet along the Z-direction, and $w_{y}$ is its width along the Y-direction. After chamfering, the effective cross-sectional area of the permanent magnet perpendicular to the magnetization direction decreases accordingly. The equivalent reluctance $R_{pm1}$ after chamfering is corrected to the following:

(4) $$R_{pm1}=\frac{t_{x}}{\mu_{1}L_{z}\left(w_{y}-\frac{1}{2t_{x}}d^{2}\right)}$$

For the equivalent reluctance of the corner region reluctance $R_{tp}$, its magnetic flux conduction path consists of two segments in series: the permanent magnet material segment and the mover silicon steel material segment. Based on the geometric characteristics of the corner region, this complex path can be simplified and approximated as an equivalent arc model. Let $\mu_{2}$ be the relative permeability of the mover silicon steel material, and $A_{tp}$ and $A_{tp1}$ be the equivalent effective areas for magnetic flux in the permanent magnet and silicon steel, respectively. The equivalent corner reluctance $R_{tp}$ can be expressed as follows:

(5) $$R_{tp}=\frac{\pi d}{{2\mu }_{1}A_{tp}}+\frac{3\pi d}{{2\mu }_{2}A_{tp1}}$$

The core focus of this study is to analyze the permanent magnet chamfering effect on magnetic field distribution. To simplify the analysis, the reluctances of other magnetic circuit components (magnetic yoke and mover silicon steel) are assumed constant and denoted as follows:

(6) $$R_{ms}+R_{gap}+R_{ss}=C$$

The total reluctance of the main magnetic circuit before chamfering $R_{main0}$ is as follows:

(7) $$R_{main0}=R_{pm0}+C$$

The corresponding main magnetic circuit flux $\Phi_{0}$ is as follows:

(8) $$\Phi_{0}=\frac{{\left(1-\alpha \right)F}_{m0}}{R_{main0}}$$

The flux in the corner shunt path $\Phi_{tp}$ is as follows:

(9) $$\Phi_{tp}=\frac{{\alpha F}_{m0}}{R_{tp}}$$

Chamfering suppresses corner flux shunting by eliminating geometric discontinuity. As chamfer depth d increases, the geometric discontinuity at the corner is replaced by a smooth transition, eliminating the original high-reluctance path. Ideally, when the chamfer depth is sufficiently large ($d\to t_{x}$), the equivalent corner reluctance $R_{tp}$ approaches infinity, and the shunt path effect becomes negligible. Under this condition, the flux in the shunt path after chamfering $\Phi_{tp1}$ can be approximated as follows:

(10) $$\Phi_{tp1}\approx 0$$

In this ideal state, the shunt coefficient α approaches 0, and the magnetomotive force of the permanent magnet acts entirely on the main magnetic circuit. The total main magnetic circuit reluctance after chamfering $R_{main1}$ is as follows:

(11) $$R_{main1}=R_{pm1}+C$$

The main flux after chamfering $\Phi_{1}$ is as follows:

(12) $$\Phi_{1}=\frac{F_{m1}}{R_{main1}}$$

The change in the main flux caused by chamfering ${\Delta \Phi }_{main}$ can be expressed as follows:

(13) $${\Delta \Phi }_{main}=\frac{F_{m}}{R_{main1}}-\frac{{\left(1-\alpha \right)F}_{m}}{R_{main0}}$$

Substituting the relevant parameters and simplifying, the flux change can be expressed as follows:

(14) $${\Delta \Phi }_{main}=F_{m}\left[\frac{1}{R_{pm1}+C}-\frac{\left(1-\alpha \right)}{R_{pm0}+C}\right]$$

Equation (14) reflects the dual effect of permanent magnet chamfering on main flux. The first term represents the flux after chamfering, and the second term represents the effective flux before chamfering. The net flux change is determined by two competing effects. Chamfering suppresses corner shunting (α→0), increasing flux. However, chamfering also increases permanent magnet reluctance ($R_{pm1}$ > $R_{pm0}$), decreasing the flux. Their relative magnitudes determine the final flux change.

According to Maxwell’s theory, the electromagnetic force $F_{e}$ acting on the air gap surface is as follows:

(15) $$F_{e}=\frac{1}{2}\frac{\left(B^{2}\right)}{\mu_{0}}A_{g}=\frac{1}{2\mu_{0}A_{g}}\Phi_{g}^{2}$$

where $A_{g}$ is the effective air gap cross-sectional area between stator and mover. $\Phi_{g}$ denotes the air-gap magnetic flux. From Equation (15), electromagnetic force is proportional to the square of air-gap magnetic flux: $F_{e}\propto \Phi_{g}^{2}$. Therefore, the net flux gain directly contributes to electromagnetic force enhancement. After derivation, the relative change in electromagnetic force before and after optimization is as follows:

(16) $$\frac{{\Delta F}_{e}}{F_{e}}=\frac{2{\Delta \Phi }_{main}}{\Phi_{0}}$$

Equation (16) provides a clear quantitative relationship, indicating that the force variation is linearly proportional to the change in the main magnetic flux.

### 2.3. Maxwell Stress Tensor Analysis: Force Directional Consistency Effect

Traditional magnetic circuit analysis methods simplify complex magnetic fields as scalars, which can effectively predict overall flux variations but cannot characterize the directional properties of magnetic field vectors and their spatial distribution non-uniformity, making it difficult to evaluate the effects of local magnetic field distortion on electromagnetic force distribution. Ideally, all electromagnetic forces should act along the mover’s motion direction, but non-uniformity and directional distortion in the actual magnetic field distribution introduce undesired force components in non-target directions, reducing force effectiveness. To overcome the limitations of the magnetic circuit method, this study employs the Maxwell stress tensor method to describe the spatial distribution of magnetic fields and their effects on electromagnetic force in vector field form. This method can reflect not only force magnitude but also force directionality and distribution characteristics.

In magnetostatic or low-frequency magnetic field environments without electric fields, the electromagnetic stress state, at any point in space, is described by the Maxwell stress tensor T, whose component form is defined as follows:

(17) $$T_{ij}=\frac{1}{\mu_{0}}\left(B_{i}B_{j}-\frac{1}{2}\delta_{ij}B^{2}\right)$$

where $T_{ij}$ is the stress tensor component representing the force density in the j-direction caused by the magnetic field in the i-direction. $B_{i}$ and $B_{j}$ are the components of the magnetic flux density vector $\vec{B}$ along the i-direction and j-direction, respectively. $\delta_{ij}$ is the Kronecker delta, which equals 1 when i = j and 0 otherwise, and $\mu_{0}$ is the vacuum permeability constant.

The squared magnitude of the magnetic flux density vector is defined as follows:

(18) $${\vec{B}}^{2}={\left|\vec{B}\right|}^{2}=B_{x}^{2}+B_{y}^{2}+B_{z}^{2}$$

According to the divergence theorem in vector analysis, the body force density vector at any point in space is as follows:

(19) $$\vec{f}=\nabla \cdot T=\frac{1}{\mu_{0}}\left[\left(\vec{B}\cdot \nabla \right)\vec{B}-\frac{1}{2}\nabla \left({\left|\vec{B}\right|}^{2}\right)\right]$$

In actual electromagnetic devices, magnetic field distribution in the air gap is influenced by both structural geometry and material properties, exhibiting significant non-uniformity and directional distortion. For analytical convenience, a two-dimensional cross-sectional model is used for simplification: the magnetic field is primarily distributed in the X-Y plane, with the Z-direction component neglected, $B_{z}$ ≈ 0. The angle between the magnetic field direction and the y-axis is defined as θ. The magnetic flux density vector can be expressed as follows:

(20) $$\vec{B}=\left(B_{x},B_{y},0\right)=\left(B_{0}\sin\theta ,B_{0}\cos\theta ,0\right)$$

where $B_{0}=\left|\vec{B}\right|$ is the magnitude of the magnetic field vector in the X-Y plane. The squared magnitude of the magnetic flux density is as follows:

(21) $${\left|\vec{B}\right|}^{2}=B_{x}^{2}+B_{y}^{2}=B_{0}^{2}\left(\sin^{2}\theta +\cos^{2}\theta \right)=B_{0}^{2}$$

This study focuses on the electromagnetic force on the air gap surface between the mover and stator. Let the normal vector of this interface be along the Y-axis direction, i.e., $\vec{n}=\left(0,1,0\right)$. Then, the force density vector $\vec{f_{s}}$ on this surface is as follows:

(22) $$\vec{f_{s}}=T\cdot \vec{n}$$

According to the stress tensor definition, the components of surface force density are as follows:

(23) $$f_{sx}=T_{xy}=\frac{1}{\mu_{0}}\left(B_{x}B_{y}\right)=\frac{B_{0}^{2}}{\mu_{0}}\sin\theta \cos\theta$$

(24) $$f_{sy}=T_{yy}=\frac{1}{\mu_{0}}\left(B_{y}^{2}-\frac{1}{2}B_{0}^{2}\right)=\frac{B_{0}^{2}}{\mu_{0}}\left(\cos^{2}\theta -\frac{1}{2}\right)$$

Therefore, the complete force density vector acting on the air gap surface is as follows:

(25) $$\vec{f_{s}}=\left(f_{sx},f_{sy},0\right)=\left(\frac{B_{0}^{2}}{\mu_{0}}\sin\theta \cos\theta ,\frac{B_{0}^{2}}{\mu_{0}}\left(\cos^{2}\theta -\frac{1}{2}\right),0\right)$$

Equation (25) shows that when the magnetic field direction deviates from the ideal Y-axis direction, an undesired tangential force component $f_{sx}$ is generated while the effective force component $f_{sy}$ in the Y-direction decreases accordingly. This occurs because magnetic field energy is redistributed across different directions, reducing force output in the target direction.

To quantitatively evaluate the consistency between the electromagnetic force vector and the target direction, a directional consistency coefficient η is defined as the ratio of the electromagnetic force component in the Y-direction to the total electromagnetic force magnitude:

(26) $$\eta =\frac{\left|F_{y}\right|}{\sqrt{F_{x}^{2}{+F}_{y}^{2}+F_{z}^{2}}}=\frac{\left|\int_{S}f_{sy}dS\right|}{\int_{S}\left|\vec{f_{s}}\right|dS}$$

where S represents the total air gap working area. The magnitude of the force density vector $\left|\vec{f_{s}}\right|$ is as follows:

(27) $$\left|\vec{f_{s}}\right|=\sqrt{f_{sx}^{2}+f_{sy}^{2}}=\sqrt{{\left(\frac{B_{0}^{2}}{{2\mu }_{0}}sin\left(2\theta \right)\right)}^{2}+{\left(\frac{B_{0}^{2}}{\mu_{0}}\left(\cos^{2}\theta -\frac{1}{2}\right)\right)}^{2}}$$

Simplifying Equation (27) yields the following:

(28) $$\left|\vec{f_{s}}\right|=\frac{B_{0}^{2}}{{2\mu }_{0}}$$

Equation (28) reveals an important physical principle: at any given point in space, the magnitude of the force density vector is determined solely by the magnetic field magnitude $B_{0}$ at that point and is independent of the magnetic field angle θ. This indicates that changing the magnetic field direction only redistributes the force density components in different directions at that point without altering the total force density magnitude. Therefore, magnetic field directional optimization cannot increase total force density but can improve the proportion of effective force component in the target direction.

### 2.4. The Unified Performance Evaluation Index System Establishment

There is an intrinsic connection between the flux variation obtained from magnetic circuit analysis and the magnetic field directional characteristics obtained from Maxwell stress analysis. Local magnetic field distortion not only causes partial flux to deviate from the main magnetic circuit but also creates magnetic field components inconsistent with the main magnetic field direction in that region, which is the primary cause of reduced electromagnetic force directional consistency.

According to magnetic field continuity conditions and Ampère’s circuital law, magnetic induction lines in the distorted region must close with the main magnetic field. At the interface between them, the magnetic induction line direction changes significantly, forming a local region with a high directional gradient. This region corresponds to the distorted area $S^{\prime }$ in the force directional consistency analysis, and relates to the shunt coefficient $\alpha_{1}$ in magnetic circuit analysis, as follows:

(29) $$\frac{S^{\prime }}{S}=k\cdot \alpha_{1}$$

where k is a geometry-related coefficient reflecting the relationship between the spatial distribution characteristics of the distorted region and the overall structure.

Based on the actual magnetic field distribution, assume that within the total effective working area S, a portion $S^{\prime }$ exhibits magnetic field directional deviation with the same deflection angle θ (as defined in Equation (20)), while the remaining area maintains an ideal magnetic field distribution. Under this assumption, the directional consistency coefficient η of the electromagnetic force is calculated using the following expression.

(30) $$\eta =\frac{\left|\int_{S-S^{\prime }}f_{sy}\left(\theta =0\right)dS+\int_{S^{\prime }}f_{sy}\left(\theta \right)dS\right|}{\int_{S-S^{\prime }}\left|\vec{f_{s}}\right|dS+\int_{S^{\prime }}\left|\vec{f_{s}}\right|dS}$$

Mathematical rearrangement and simplification of Equation (30) yield the following:

(31) $$\eta =1-\frac{S^{\prime }}{S}\left(1-\left(\cos2\theta \right)\right)=1-k\cdot \alpha_{1}\left(1-\left(\cos2\theta \right)\right)$$

Based on flux variation analysis and force directional consistency variation analysis, a comprehensive electromagnetic performance evaluation index is established. The comprehensive electromagnetic performance index P is defined as follows:

(32) $$P=F_{e}\cdot \eta$$

This index considers both electromagnetic force magnitude and directional effectiveness. Correspondingly, the performance change rate can be expressed as a linear superposition of the flux variation effect and the force directional consistency variation effect:

(33) $$\frac{\Delta P}{P}=\frac{{\Delta F}_{e}}{F_{e}}+\frac{\Delta \eta }{\eta_{0}}$$

where the relative change in electromagnetic force magnitude is as follows:

(34) $$\frac{{\Delta F}_{e}}{F_{e}}=\frac{2{\Delta \Phi }_{main}}{\Phi_{0}}=\frac{2\alpha }{1-\alpha }\left[1+\frac{R_{pm0}-R_{pm1}}{R_{pm0}+C}\right]$$

The absolute change in force directional consistency coefficient is as follows:

(35) $$\Delta \eta =k\cdot \alpha_{1}\left(1-\left(\cos2\theta \right)\right)$$

Substituting the electromagnetic force magnitude change and force directional consistency coefficient change into the comprehensive performance expression yields a unified expression for permanent magnet geometric optimization effects:

(36) $$\frac{\Delta P}{P}=\frac{2\alpha }{1-\alpha }\left[1+\frac{R_{pm0}-R_{pm1}}{R_{pm0}+C}\right]+\frac{k\cdot \alpha_{1}\left(1-\left(\cos2\theta \right)\right)}{\eta }$$

Equation (36) expresses the performance change as the sum of two terms. The first term originates from flux variation, reflecting the combined effect of suppressing corner shunting and increasing permanent magnet reluctance, and the second term originates from improved magnetic field directional consistency, reflecting the positive impact of eliminating magnetic field distortion on force directional characteristics. The relative contributions of the two terms vary with chamfer depth: the first term dominates at small chamfer depths, while the second term becomes more important at large chamfer depths.

### 2.5. Parametric Analysis of the Theoretical Model

Based on the unified performance evaluation theoretical model, Equation (36) is used to quantitatively predict the permanent magnet chamfering optimization effect. To maintain consistency with subsequent simulation and experimental conditions, the permanent magnet thickness is set to $t_{x}=8\;mm$, with chamfer depth d varying continuously within the 0–8 mm range.

The flux shunt coefficient $\alpha_{1}$ is derived from the geometric area loss due to chamfering. Assuming uniform magnetization, the magnetic flux through the permanent magnet cross-section is proportional to its area. Chamfering reduces the effective flux-supplying area, and the “lost” flux is redistributed into the working air-gap via alternative magnetic paths, forming the shunted flux. Thus, the maximum flux shunt ratio theoretically approximates the maximum area-loss ratio.

For a 45° chamfer, the maximum area loss ratio $R_{area}$ occurs when the chamfer depth equals the magnet thickness $d=t_{x}=8\;mm$.

(37) $$R_{area}=\frac{t_{x}}{2w_{y}}$$

Substituting the geometric dimensions gives $R_{area}$ = 0.167. This value represents the theoretical upper limit of $\alpha_{1}$. In practice, flux redistribution is not ideal due to fringing effects and leakage. Therefore, a conservative value of $\alpha_{1}$ = 0.11 is adopted. This choice ensures physical feasibility while accounting for practical losses.

The geometric correlation coefficient k quantifies how effectively local magnetic improvement near the chamfer translates into overall performance gain. Ideally, if the improvement propagated without loss, k would equal 1. In reality, edge effects, field decay, and three-dimensional leakage reduce its value, so k < 1. Following a conservative engineering approach, k = 0.8 is adopted in this study.

Precise reluctance calculation would require resolving complex three-dimensional boundary conditions and nonlinear field distributions. Here, engineering-based approximate values are used to capture trends and parametric relationships rather than to pursue absolute numerical accuracy.

**Figure 3:**
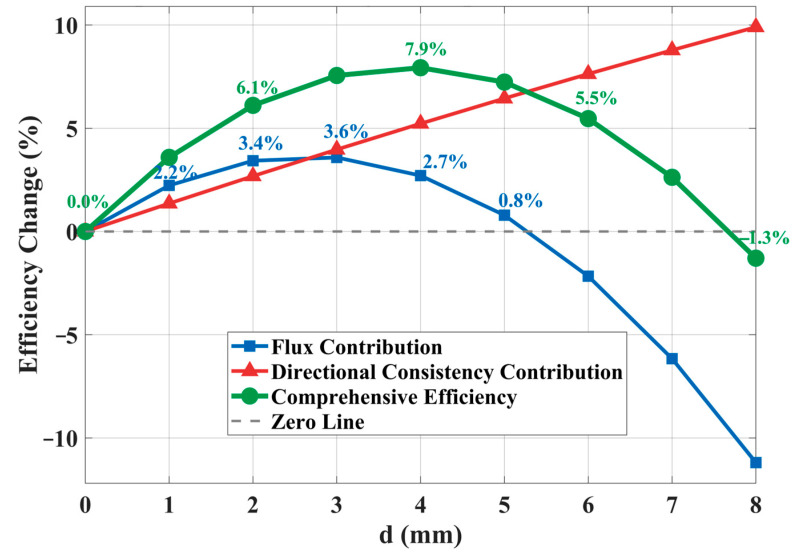
Theoretical prediction of chamfer optimization effects.

Figure 3 presents the theoretical predictions of the model, providing a baseline for subsequent numerical simulation and experimental verification. The blue curve represents the first term of in (36) (flux contribution), the red curve shows the second term (directional consistency contribution), and the green curve corresponds to their sum (overall performance change).

The flux contribution (blue curve) rises with chamfer depth up to 3 mm, peaks, and subsequently falls to a lower value at 8 mm. This trend captures the dual role of chamfering: at shallow depths, it mainly suppresses flux shunting at corners, thereby increasing useful flux; beyond a certain depth, the reduction in permanent-magnet cross-section raises magnetic reluctance and reduces flux. The observed peak aligns with theoretical expectation, confirming the internal consistency of the model. In contrast, the directional consistency contribution (red curve) increases monotonically, reaching about 10.0% at 8 mm. This steady gain results from the progressive smoothing of geometric discontinuities, which continuously reduces the magnetic-field deflection angle. The stronger growth of this term highlights that chamfering influences performance more substantially through field direction improvement than through flux enhancement alone.

The overall performance curve (green) rises within the range of 0–4 mm, reaches a peak of approximately 7.9% near 4 mm, and then gradually declines to −1.3% at 8 mm. This curve reflects the competition between two effects. From 0 to 4 mm, both flux enhancement and directional improvement contribute positively, resulting in rapid performance growth. In the moderate chamfer region (4–6 mm), the continuing increase in directional consistency still outweighs the declining flux contribution, maintaining a net positive gain. For larger chamfers (d > 6 mm), however, the negative effect of flux loss gradually surpasses the positive effect of directional improvement, causing the overall performance to decrease. Notably, when d exceeds 7 mm, the overall performance turns negative, indicating that excessive chamfering degrades system performance.

This variation characteristic indicates the existence of a theoretically optimal chamfer depth range. Within this range, the positive effects of flux enhancement and directional consistency improvement can effectively superpose while avoiding negative impacts from excessive geometric modification.

### 2.6. Parameter Sensitivity Analysis

**Figure 4:**
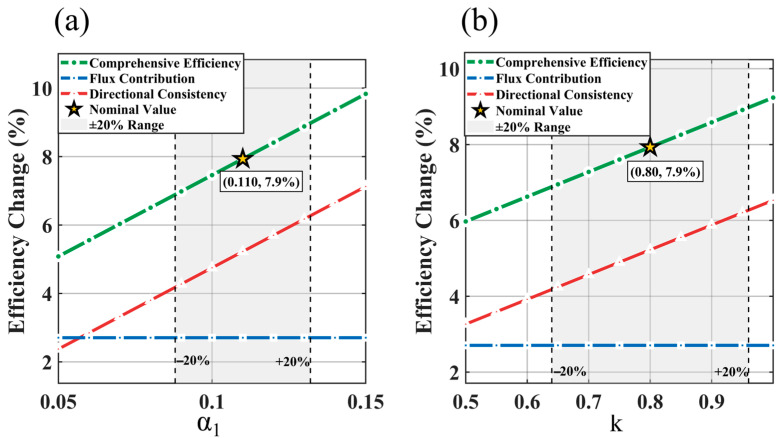
Sensitivity Analysis. (**a**) Sensitivity to $\alpha_{1}$; (**b**) sensitivity to k.

A sensitivity analysis is conducted at the optimal chamfer depth d = 4 mm to evaluate the influence of parameter uncertainty. With geometric parameters fixed, the flux shunt coefficient $\alpha_{1}$ and the geometric correlation coefficient k are varied within ±20% of their nominal values, and the resulting impact on the overall efficiency is examined. The results are shown in Figure 4, where the blue dashed line represents the flux contribution, the red dashed line denotes the directional consistency contribution, and the green solid line corresponds to the overall efficiency. The yellow star marks the nominal parameter values ($\alpha_{1}$ = 0.11, k = 0.8 and efficiency 7.9%), and the gray shaded areas indicate the ±20% variation ranges.

In Figure 4a, the flux contribution remains constant at 2.7 percent, independent of α_1_, confirming its geometric nature. The directional consistency contribution increases with $\alpha_{1}$, indicating improved field uniformity. Within the ±20 percent range of $\alpha_{1}$ (0.088 to 0.132), the overall efficiency varies from 6.8 percent to 8.7 percent, corresponding to a sensitivity coefficient of 24 percent.

In Figure 4b, we present a similar pattern. The flux contribution is constant, while the directional consistency contribution rises with k. Over the ±20% interval (0.64–0.96), the overall efficiency ranges from 7.3% to 8.9%, yielding a sensitivity coefficient of about 20%.

Even with ±20% parameter uncertainty, the efficiency at d = 4 mm stays above 6.8%, proving that chamfering remains beneficial. The constant flux contribution verifies the model’s physical consistency. This analysis assesses the impact of parameter errors—not re-optimization. The nominal values $\alpha_{1}$ = 0.11, k = 0.80) are based on theory and conservatism. The results confirm that the optimization conclusions are robust to ±20% parameter variations, supporting the model’s engineering applicability.

## 3. Finite Element Simulation Analysis

### 3.1. Establishment of Simulation Model

**Figure 5:**
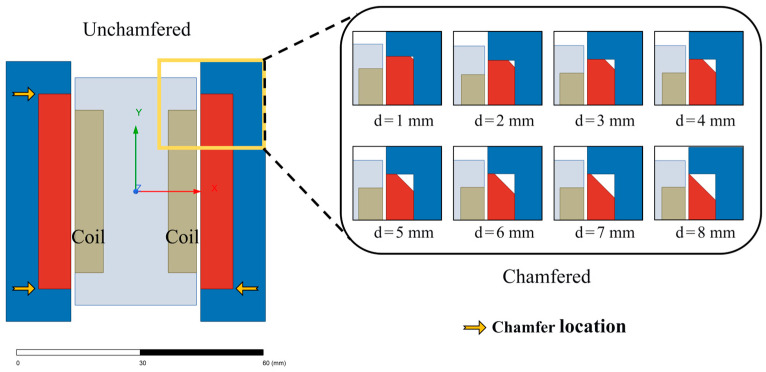
Two-dimensional finite element simulation model.

To verify the theoretical analysis, a two-dimensional finite element model of the electromagnetic vibration absorber core structure was established using ANSYS Maxwell electromagnetic field simulation software (Version 2024 R1, ANSYS, Inc., Canonsburg, PA, USA). Parametric scanning of chamfer depth d was performed in the 0–8mm range with a step size of 1 mm. The chamfer geometric angle was uniformly set to 45°, consistent with theoretical analysis. The geometric structures corresponding to different chamfer depths are shown in Figure 5.

This study employs a 2D simulation approach, appropriate for capturing the relative performance trends of chamfer geometry. Although 3D effects like end-region flux leakage and axial non-uniformity are not explicitly modeled, they influence all chamfer depths uniformly, primarily altering the absolute force magnitude rather than the optimal depth selection.

### 3.2. Flux Characteristic Analysis

**Figure 6:**
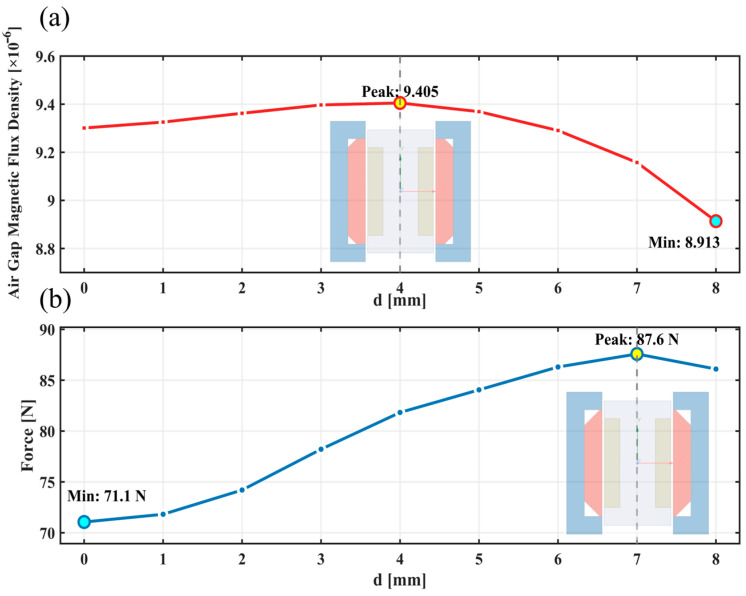
Effect of chamfer depth on key performance parameters. (**a**) Air gap magnetic flux density variation; (**b**) electromagnetic force variation.

Through steady-state electromagnetic field simulation, information on flux distribution, magnetic field vectors, and local magnetic fields was extracted. Electromagnetic force calculation employed the Maxwell stress tensor method, with the integration path set at the air gap centerline to ensure consistency and comparability of results. Simulation results are shown in Figure 6.

In Figure 6a, we show the variation in the line-integrated magnetic flux density at the center of the working air-gap with respect to the chamfer depth. The simulation results show that the effective working flux reaches its peak value at d = 4 mm and then decreases. The theoretical prediction peaks at d = 3 mm (Figure 3), with a difference of only 1 mm, and the trend is consistent. This verifies the dual effect of chamfering on magnetic flux: a moderate chamfer increases effective flux, whereas an excessive depth reduces it.

In Figure 6b, we show the variation in electromagnetic force with chamfer depth. Unlike flux density variation, electromagnetic force continues to increase, reaching a maximum of 87.6 N at d = 7 mm, representing a 23.2% force enhancement compared to 71.1 N without chamfering. At d = 8 mm, the electromagnetic force slightly decreases to 85.3 N but remains significantly higher than the initial value.

A comparison between the simulation results (Figure 6a,b) and the theoretical prediction (Figure 3) shows that both reveal a separation between the flux peak and the performance peak. The magnetic flux reaches its maximum at a chamfer depth of 4 mm, while the thrust performance continues to increase and attains its optimum at 7 mm. This observation supports the core conclusion of the theoretical model, that thrust improvement stems primarily from enhanced field directionality, not from an increase in flux. The theoretical model predicts a monotonic rise in directional contribution with chamfer depth, which aligns with the simulation trend.

Regarding the optimum depth, the theoretical model and the simulations exhibit a numerical discrepancy. Nevertheless, both confirm that the dominant factor in performance improvement is the enhancement of field directionality.

### 3.3. Field Distribution Characteristic Analysis

To gain deeper insight into the specific effects of chamfering on magnetic field distribution, a before-and-after comparison of electromagnetic field distribution is conducted using 4 mm chamfering as an example. The 4 mm chamfer depth is selected based on two main considerations. First, it corresponds to the peak of overall performance predicted by the theoretical model. Second, the simulation shows that the slope of the flux-density curve near this depth approaches zero, indicating low sensitivity to small variations in chamfer geometry. This feature helps improve process tolerance and reduces the impact of manufacturing deviations on performance.

By extracting key physical quantities such as magnetic field gradient, flux density distribution, and electromagnetic force vector, the effectiveness of geometric optimization is comprehensively evaluated.

**Figure 7:**
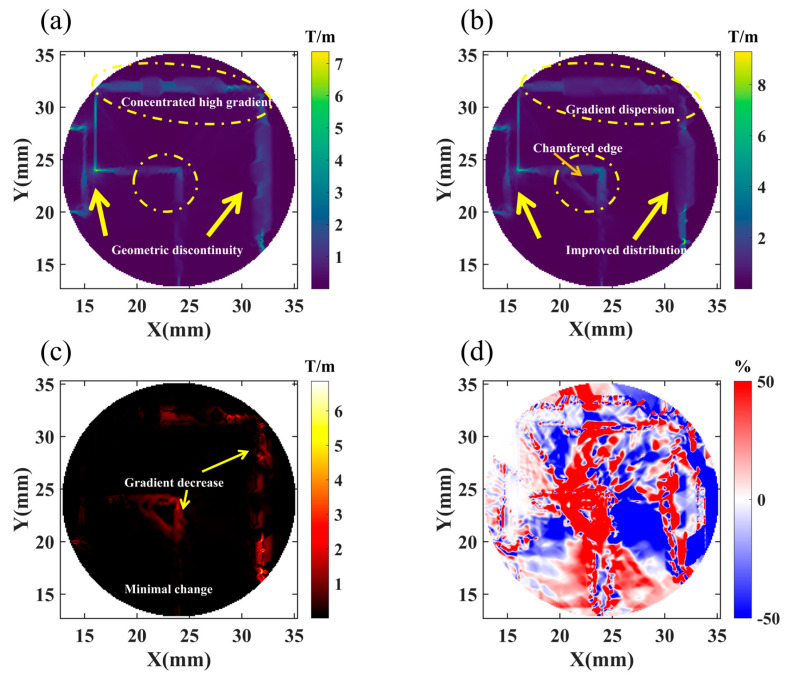
Comparative analysis of magnetic field gradient distribution. (**a**) Gradient distribution before chamfering; (**b**) gradient distribution after chamfering; (**c**) absolute difference; (**d**) relative difference.

Figure 7 shows the effect of chamfering on the local magnetic field gradient distribution. The magnetic field gradient reflects the spatial rate of change in the magnetic field, and its magnitude directly characterizes the uniformity of magnetic field distribution, serving as an important indicator for evaluating geometric optimization effectiveness.

Figure 7a shows the magnetic field gradient distribution before chamfering. The region marked by the yellow dashed circle (at the geometric edge between permanent magnet and mover silicon steel) exhibits an obviously high-gradient concentration, with colors shifted toward the high end of the color scale, indicating severe spatial variation in the magnetic field. This gradient concentration originates from the sharp bending of magnetic induction lines caused by geometric discontinuity, with the yellow arrow indicating the primary gradient direction.

Figure 7b shows the gradient distribution characteristics after 4 mm chamfering. Comparing with Figure 7a, the high-gradient concentration at the original geometric edge is significantly alleviated, with color distribution tending toward the mid-to-low range of the color scale, indicating reduced gradient peaks. Meanwhile, the gradient distribution becomes more spatially dispersed; although the influence range of high-gradient regions expands somewhat, the degree of local abruptness is significantly weakened.

Figure 7c shows the absolute difference distribution of the magnetic field gradient before and after chamfering. Color variations intuitively display the specific regions and degrees of chamfering effects on the magnetic field gradient. Red regions indicate locations where the gradient significantly decreases after chamfering, mainly distributed at the sharp corners of the original geometric edges, which are precisely the areas directly affected by chamfering. Black background regions indicate very small or near-zero gradient changes, demonstrating that the chamfering effect has obvious spatial locality, primarily concentrated within the vicinity of geometric modification.

Figure 7d quantifies the gradient variation magnitude in the form of relative difference percentages, with red and blue colors distinguishing regions of gradient decrease and increase. Red regions correspond to original high-gradient concentration areas, indicating that chamfering effectively improves local magnetic field distribution; blue regions are located near the chamfered surface, showing slight gradient increases resulting from magnetic field redistribution. Overall, chamfering significantly reduces the maximum gradient value and makes gradient distribution more uniform, helping to alleviate local magnetic saturation and leakage flux while improving magnetic field utilization efficiency.

**Figure 8:**
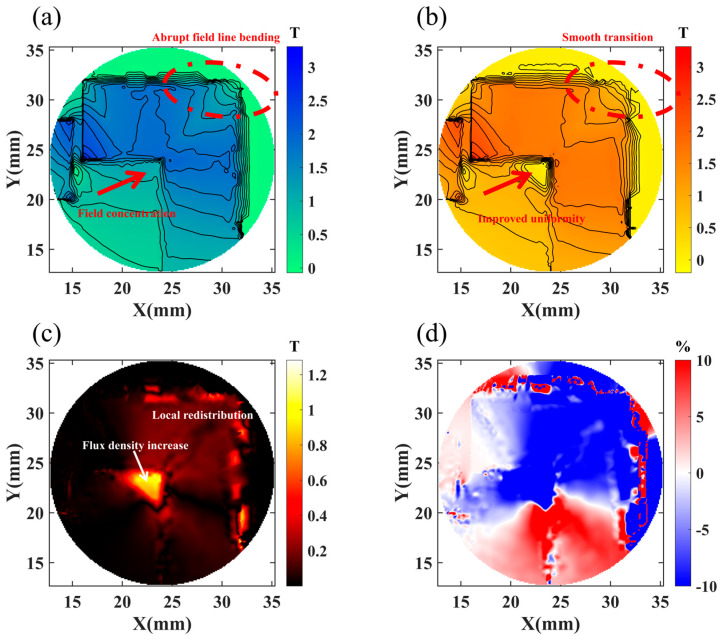
Comparative analysis of magnetic flux density distribution. (**a**) Magnetic field distribution before chamfering; (**b**) magnetic field distribution after chamfering; (**c**) absolute difference; (**d**) relative difference.

Figure 8 reflects the effect of chamfering on the spatial distribution of flux density. In Figure 8a, we show the magnetic field distribution characteristics before chamfering. From the magnetic induction line trajectories, obvious sharp bending and local concentration can be observed near the permanent magnet sharp edges (region marked by red dashed circle), with red arrows indicating the primary regions of magnetic field concentration. The air gap region exhibits a non-uniform flux density distribution, with low-density areas (blue, green) coexisting with high-density concentration zones. This non-uniform distribution directly manifests magnetic field distortion caused by geometric discontinuity.

Figure 8b shows the improved magnetic field distribution after 4 mm chamfering. Comparing with Figure 8a, chamfering produces smoother and more gradual magnetic induction line transitions in the geometrically modified region, alleviating the original sharp bending. The region indicated by the red arrow shows improved magnetic field distribution uniformity. From the color distribution, orange-yellow tones occupy a larger proportion overall, indicating a more uniform flux density distribution within the working air gap with reduced spatial variation gradients. The smooth transition of magnetic induction lines indicates that chamfering effectively eliminates magnetic field abruptness caused by geometric discontinuity, benefiting flux utilization efficiency.

In Figure 8c, we show the absolute difference distribution of flux density before and after chamfering. Black regions indicate minimal change, while red to yellow represents density increase. The distribution characteristics show that chamfering effects are mainly concentrated in two aspects: first, flux density redistribution occurs in the original sharp edge regions; second, flux density slightly increases in the air gap center region.

Figure 8d quantifies the flux density variation magnitude in relative difference percentages. Red regions represent locations where flux density relatively increases, mainly distributed in the air gap center region and near the chamfered surface, with relative increases reaching approximately +10%. Blue regions represent locations where flux density relatively decreases, mainly distributed in local areas of the original geometric edges, with relative decreases of approximately −10%. White or light-colored regions represent locations with very small relative changes. This distribution indicates that chamfering redistributes flux from edge concentration zones to effective working regions, achieving overall uniformity that benefits magnetic field utilization efficiency and electromagnetic force output. Notably, despite local increases and decreases, the overall flux density variation range is controlled within ±10%, indicating that chamfering is a moderate and effective optimization method that does not cause drastic magnetic circuit changes.

**Figure 9:**
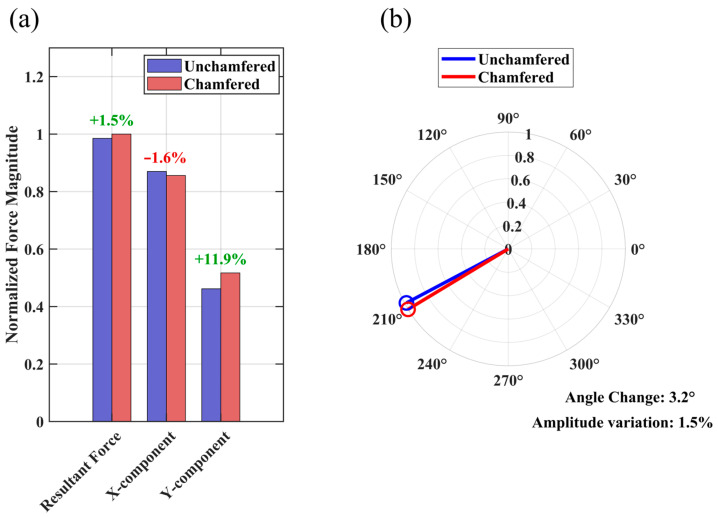
Analysis of electromagnetic force vector characteristics. (**a**) Comparison of force components in different directions; (**b**) resultant force vector variation.

In Figure 9, we systematically demonstrate the effect of chamfering on electromagnetic force vector characteristics. The force component analysis results in Figure 9a show that after chamfering, the X-direction force component decreases by 1.6%, the Y-direction force component increases by 11.9%, and the resultant force magnitude increases by 1.5% overall. This beneficial redistribution of force components indicates that chamfering effectively improves the directional characteristics of electromagnetic force. The polar coordinate analysis in Figure 9b shows a 3.2° angular change in resultant force direction with a 1.5% magnitude change. Although the absolute angular change value is relatively small, this systematic directional improvement has engineering significance for effective electromagnetic force utilization.

### 3.4. System Performance Verification

To comprehensively evaluate the practical engineering applicability of the chamfering optimization scheme, a comparative analysis of electromagnetic force characteristics under different current conditions was conducted. Parametric scanning of coil excitation current from 1 A to 5 A was performed to study the variation patterns of electromagnetic force at different operating points.

**Figure 10:**
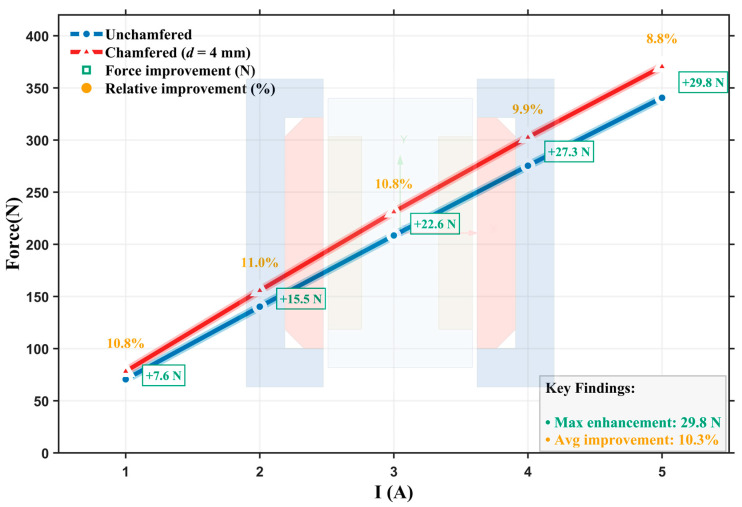
Comparison of electromagnetic force characteristics under different current conditions.

In Figure 10, we show the electromagnetic force comparison results before and after chamfering in the 1–5 A current range. From the current-force characteristic curves, positive improvements are observed for the optimized structure with chamfering under all tested current conditions, with absolute increments increasing from 7.6 N at 1 A to 29.8 N at 5 A. The relative improvement rate varies in the 8.8–11.0% range, with an average improvement rate of approximately 10.3%. This indicates that the chamfering optimization effect is positively correlated with system operating intensity. Additionally, electromagnetic force under both structures exhibits good linearity, demonstrating that chamfering optimization does not alter the fundamental electromagnetic characteristics of the system.

**Figure 11:**
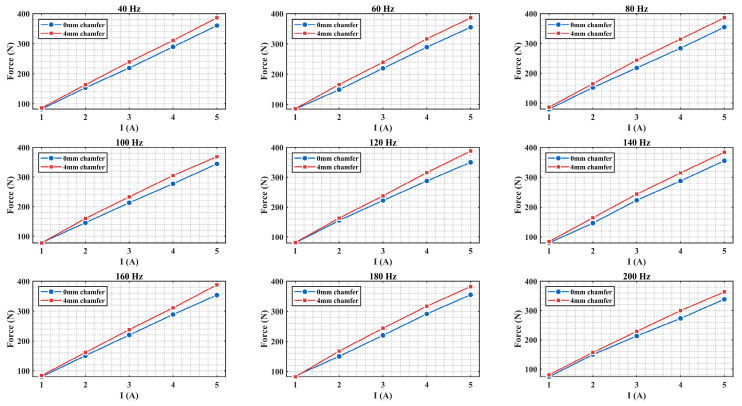
Current-force characteristic curves at different frequencies (simulation).

To verify the effectiveness of chamfering optimization technology under dynamic conditions, a transient electromagnetic simulation analysis was conducted. Figure 11 shows the electromagnetic force output comparison results for structures without chamfering and with 4 mm chamfering under different current excitations in the 40–200 Hz frequency range.

Transient simulation results indicate that under all tested frequency conditions, the electromagnetic force output of the 4 mm chamfered structure is significantly higher than that of the non-chamfered structure, with both structures maintaining good current-force linearity. This result is consistent with the conclusions from the steady-state simulation analysis, further confirming the effectiveness of the chamfering optimization method.

**Figure 12:**
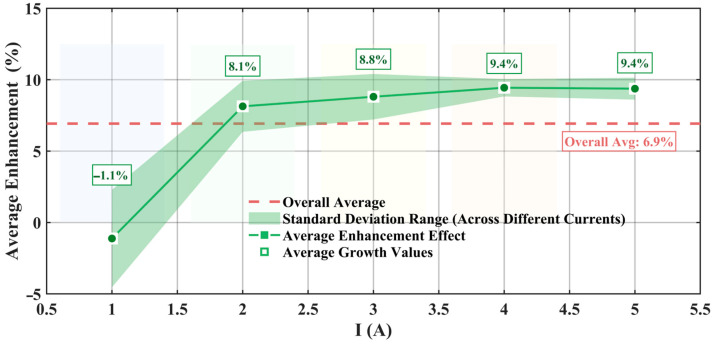
Average enhancement effect under different currents (simulation).

Statistical analysis in Figure 12 shows the variation pattern of the chamfering optimization effect with excitation current. As the current gradually increases, the electromagnetic force progressively improves, with an overall average enhancement effect reaching 6.9%, demonstrating the engineering value of chamfering optimization technology.

## 4. Experimental Verification

### 4.1. Experimental System Setup

**Figure 13:**
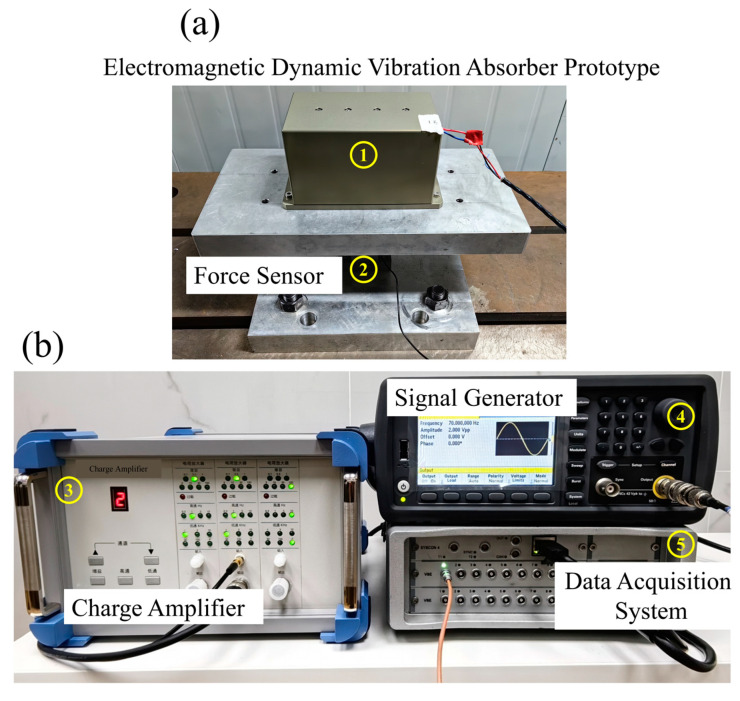
Force testing system. (**a**) Test platform; (**b**) data acquisition system.

Based on theoretical analysis and simulation verification results, an experimental prototype with a 4 mm chamfer depth was fabricated, and a complete electromagnetic vibration absorber system test platform was constructed for experimental verification. The experimental setup is shown in Figure 13.

The 4 mm chamfer depth is selected for two primary reasons. First, it coincides with the peak in overall performance predicted by the theoretical model (Figure 3), allowing a direct validation of the model. Second, the simulation results show that near this depth, the system exhibits relatively low sensitivity to chamfer variations (Figure 6a). This characteristic provides practical benefits, as it allows greater process tolerance and reduces the effect of manufacturing variations on experimental outcomes.

The primary aim of this experiment is to validate the proposed “magnetic field directional consistency” metric and the effectiveness of the collaborative design methodology. Validation was achieved by obtaining a specific design point—one that is both engineering-feasible and significantly outperforms the no-chamfer baseline. To accomplish this, the theoretically predicted optimal point (chamfer depth of 4 mm) was selected for experimental verification. The successful attainment of performance at this specific point demonstrates the validity and utility of the presented method. Once the fundamental validity of the method is established, further application-oriented research can be conducted to characterize the continuous performance trend across the entire theoretically optimal interval.

The experimental test system consists of the following equipment (see Figure 13): Device 1 is the electromagnetic vibration absorber prototype under test, Device 2 is a high-precision force sensor, Device 3 is a charge amplifier, Device 4 is a signal generator, and Device 5 is a high-speed data acquisition system. Under identical operating conditions, electromagnetic force performance tests were conducted on electromagnetic vibration absorber prototypes with and without chamfering.

Experimental testing employed a quasi-static loading method, measuring corresponding electromagnetic force output by precisely controlling excitation current amplitude and frequency. To ensure experimental result reliability, each operating condition point was tested three times, with the average taken as the final result. Experimental environment temperature was controlled at 20 ± 2 °C, with relative humidity controlled at 50 ± 5%.

### 4.2. Experimental Results Analysis

**Figure 14:**
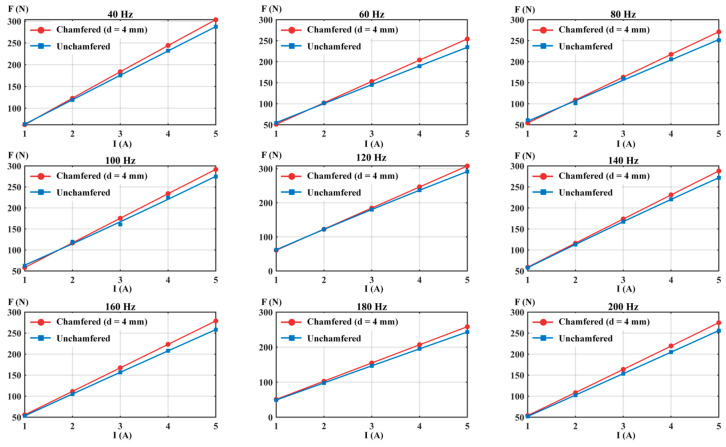
Current-force characteristic curves at different frequencies (experimental).

Electromagnetic force performance test results are shown in Figure 14.

Figure 14 shows the current-force output relationship of prototypes before and after chamfering in the 40–200 Hz frequency range. Experimental results indicate that under all tested frequency and current combinations, the chamfered optimized structure exhibits higher force output than the non-chamfered structure, confirming simulation analysis predictions. From the linearity analysis of current-force characteristics, both structures maintain good linear characteristics at all frequencies, demonstrating that chamfering optimization does not alter the fundamental electromagnetic characteristics of the system and preserves the linear response characteristics of the structure.

**Figure 15:**
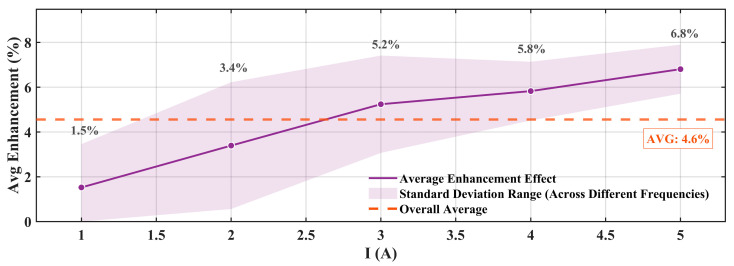
Average enhancement effect under different currents (experimental).

Systematic statistical analysis was performed on all experimental test data to quantitatively evaluate the overall chamfering optimization effect. Figure 15 shows the statistical results of force output improvement effects under different currents.

Figure 15 results show that the enhancement effect of chamfering optimization exhibits an increasing trend with current, progressively improving from 1.5% at 1 A to 6.8% at 5 A, with an average improvement effect of 4.6%. The purple shaded region in the figure represents the standard deviation range of test results at different frequencies under the same current conditions, and the width of this region directly reflects the frequency stability of the chamfering optimization effect.

From the analysis in Figure 15, the following can be observed: in the low-current region (1–3 A), the standard deviation is relatively large, and the shaded area is wider, indicating a certain degree of variability in test results across different frequencies under lower excitation intensity conditions. This is mainly because signal strength is relatively weak under low-current conditions, and the relative impact of measurement system noise and environmental interference on measurement results is more significant. As the current gradually increases to the medium-to-high current region (3–5 A), the standard deviation significantly decreases, and the shaded area correspondingly narrows. This is because under stronger magnetic field conditions, the system’s electromagnetic response is more stable and less affected by external interference factors.

### 4.3. Comparison Between Experimental Results and Theoretical Predictions

Through comparative analysis, experimental results show good consistency with theoretical model predictions in overall variation trends, although certain numerical differences exist. The theoretical model predicts a performance improvement of approximately 6.9% for a 4 mm chamfer depth, while the actual experimental measurement is 4.6%.

The main reasons for experimental values being lower than theoretical predictions include the following: simplified assumptions adopted during theoretical model establishment, neglecting actual factors such as end effects, eddy current losses, and hysteresis losses; batch-to-batch differences and non-uniformity in material properties; impacts of machining and assembly precision on performance; environmental factors and measurement errors during experimental testing. Despite numerical differences, the magnitude of discrepancy is within the acceptable range for engineering predictions, verifying the effectiveness of the chamfering optimization method and the practicality of the theoretical model.

## 5. Conclusions

This study proposes a structural optimization method based on permanent magnet chamfering to address the magnetic field distribution non-uniformity caused by geometric discontinuity in electromagnetic vibration absorbers. Through establishing a unified theoretical analysis model, the influence mechanisms of chamfer geometric parameters on magnetic field distribution and electromagnetic force output characteristics are systematically analyzed.

The main research conclusions are as follows:

(1) A dual-effect theoretical analysis framework for electromagnetic vibration absorber chamfering optimization was established. Through magnetic circuit analysis and Maxwell stress tensor analysis, the flux enhancement effect and force directional consistency improvement effect produced by chamfering were revealed, and a unified mathematical model was established.

(2) The effect transition pattern during chamfering optimization was discovered. At small chamfer depths, the flux enhancement effect dominates, while at large chamfer depths, force directional consistency improvement becomes the primary contribution. The competition and synergy between these two effects determine the overall optimization effectiveness.

(3) The asynchronous phenomenon between flux density variation and electromagnetic force output was revealed. In the 4–7 mm chamfer depth range, flux density decreases while electromagnetic force continues to increase, demonstrating that electromagnetic force output depends not only on flux density magnitude but is more influenced by magnetic field directional distribution characteristics.

(4) The engineering effectiveness of the chamfering optimization method was verified. Experiments conducted in the 40–200 Hz frequency range and 1–5 A current conditions demonstrate that the 4 mm chamfered optimized structure achieves an average output force improvement of 4.6%, effectively improving electromagnetic force output performance without changing the device’s external dimensions.

The optimization method and performance metrics proposed in this study have direct implications for enhancing the overall performance of electromagnetic vibration control systems. The increase in electromagnetic force amplitude provides the system with a greater control force margin, enabling it to handle higher-intensity vibrational disturbances. Simultaneously, the improvement in magnetic field directional consistency ensures that the electromagnetic force is applied more accurately along the target direction, which not only increases the actuation efficiency but also significantly reduces additional vibrations and system interference caused by undesired lateral force components. This co-optimization of “force amplitude” and “force direction” establishes a critical device-level foundation for building more stable, robust, and responsive active vibration control systems.

This research has reference value for electromagnetic actuator design in space-constrained environments, and the established dual-effect theoretical analysis framework provides theoretical guidance for geometric optimization of other types of electromagnetic structures.

## Data Availability

The datasets generated and/or analyzed during the current study are not publicly available due to confidentiality restrictions under legally binding agreements. However, processed data supporting the key findings of this study are available from the corresponding author upon reasonable request.
